# Supplementary material for: Predictors and Profile of Severe Infectious Complications in Multiple Myeloma Patients Treated with Daratumumab-Based Regimens: A Machine Learning Model for Pneumonia Risk
Source: Cancers (Basel). 2024 Nov 3;16(21):3709. doi: 10.3390/cancers16213709 (PMC11545561; doi:10.3390/cancers16213709)
Supplement: Supplementary file 1 [file cancers-16-03709-s001.zip › Supplementary Materials 31.10.2024.pdf]

Supplementary File S1. The dataset used for the development of a classification model to predict pneumonia in patients treated with Daratumumab-based regimens. The dataset is uploaded in a separate Excel file.

Supplementary File S2. The developed classification model using WEKA software. The file is uploaded separately.

Supplementary File S3. The developed classification models- A Stochastic Gradient Boosting (GB) and Random Forest (RF)- for pneumonia were constructed in Python 3.11.4 using the scikit-learn package version 1.5.2. The file is uploaded separately.

Supplementary Table S1. Univariate logistic regression analysis of factors associated with pneumonia occurrence in multiple myeloma (MM) patients treated with daratumumab-based regimens.

Variable	OR	95% CI		p-value
		Lower	Upper	
**ECOG>1**	4.781	1.737	13.158	0.0024
**PDW (fL)**	0.607	0.429	0.859	0.0048
**Hemoglobin (g/dL)**	0.796	0.645	0.982	0.0331
**AST (U/L)**	1.010	0.995	1.025	0.1936
**Relapsed disease**	0.543	0.212	1.388	0.2022
**Neutrophil Count (10^9/L)**	1.148	0.915	1.441	0.2321
**Calcium (mmol/L)**	2.536	0.479	13.426	0.2737
**M Protein (g/L)**	1.010	0.989	1.032	0.3599
**Stage 3 ISS/R-ISS***	1.545	0.586	4.073	0.3787
**Age>65**	1.518	0.586	3.934	0.3898
**Platelets (10^9/L)**	0.998	0.992	1.003	0.4235
**Bilirubin (mg/dL)**	0.495	0.086	2.859	0.4316
**RDW-SD (fL)**	1.017	0.974	1.061	0.4494
**ALT (U/L)**	1.005	0.990	1.021	0.5067
**Urea (mmol/L)**	1.007	0.986	1.028	0.5393
**Gender-Male**	1.325	0.511	3.435	0.5625
**Gamma Globulins (g/L)**	0.962	0.789	1.173	0.7021
**Lymphocyte Count (10^9/L)**	0.963	0.621	1.492	0.8661
**CRP (mg/L)**	0.998	0.976	1.021	0.8722
**Albumin (g/L)**	1.007	0.928	1.092	0.8744
**Creatinine (mg/dL)**	0.955	0.503	1.811	0.8876
**WBC (10^9/L)**	1.005	0.841	1.200	0.9584

* - For NDMM, the R-ISS is provided, while for RRMM, the ISS is used due to the absence of cytogenetic data.

ALT - alanine aminotransferase; AST - aspartate aminotransferase; CRP - C-reactive protein; ECOG - Eastern Cooperative Oncology Group; ISS- International Staging System; OR- odds ratio; PDW - platelet

distribution width; RDW-SD - red cell distribution width - standard deviation; R-ISS- Revised International Staging System; WBC - white blood cell count.

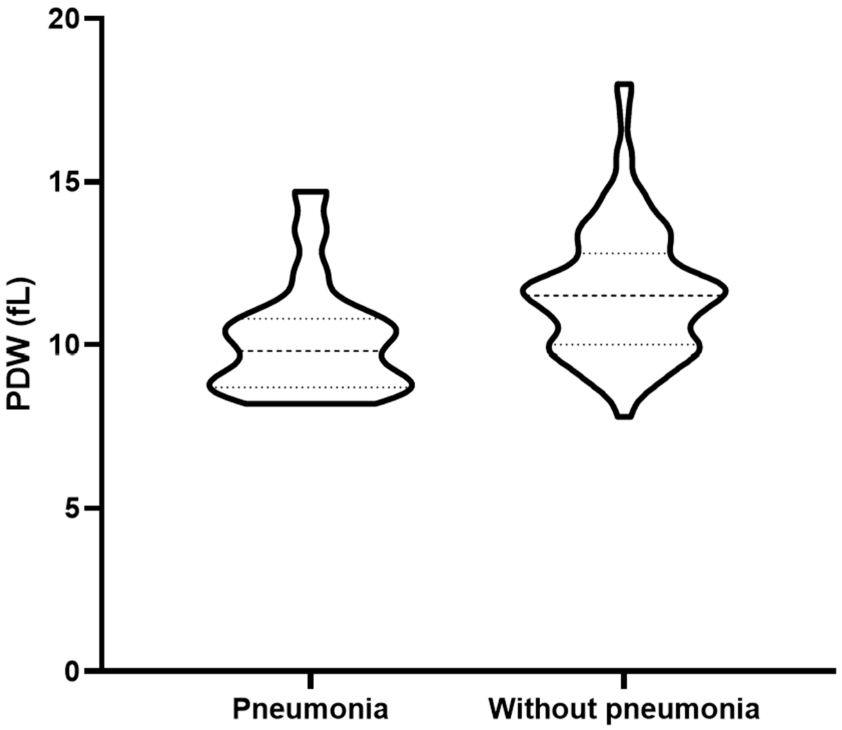

Supplementary Figure S1. Comparison of PDW Between Pneumonia and Non-Pneumonia Groups. We found a statistically significant difference in PDW between the pneumonia group (median 9.8 fL, IQR: 8.7–10.8 fL) and the non-pneumonia group (median 11.5 fL, IQR: 10.0–12.8 fL, p = 0.0011).

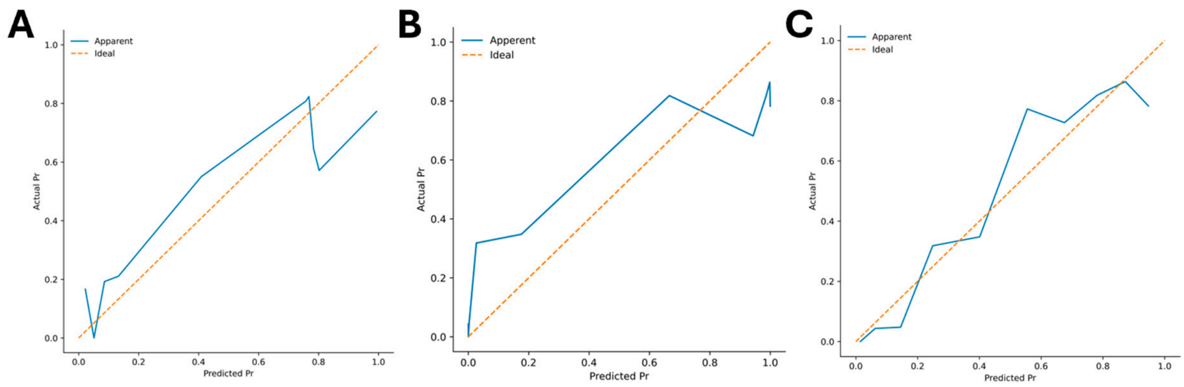

Supplementary Figure S2. Calibration plots for developed classification models. A. J48 decision tree; B. gradient boosting; C. random forest.
